# Serum IL-35 is decreased in overweight patients with rheumatoid arthritis: its correlation with Th1/Th2/Th17-related cytokines

**DOI:** 10.1186/s12865-021-00431-x

**Published:** 2021-06-27

**Authors:** Yuxuan Li, Yang Jie, Xiaofei Wang, Jing Lu

**Affiliations:** 1 Department of Rheumatology and Immunology, Shengjing Hospital of China Medical University, No. 36 San Hao Street, Heping District, Shenyang, 110004 PR China; 2 Department of Rheumatology and Immunology, The First Affiliated Hospital of China Medical University, 155 Nanjing North Street, Heping District, Shenyang, 110001 PR China

**Keywords:** Interlukin-35, Th1/Th2/Th17 cytokines, Rheumatoid arthritis, Obesity

## Abstract

**Background:**

Obesity is correlated with worse drug responses and high disease activity in patients with rheumatoid arthritis (RA). Interleukin (IL)-35 is a novel anti-inflammatory cytokine that mainly produced by regulatory T (Treg). This study was performed to analyze whether IL-35 was correlated with obesity in RA and investigate the correlation between other Th1/Th2/Th17-related cytokines and obesity in RA.

**Results:**

The serum IL-35 level was analyzed in RA (*n* = 81) and healthy donors (*n* = 53) by ELISA assay, and was compared between three groups (body mass index (BMI) < 18.5,≥18.5 to 25, > 25). Serum cytokines including IL-2, IL-4, IL-10, IL-17, INF-γ, TNF-α levels were measured using Flowcytometry assay. Clinical information was extracted from medical records. Serum IL-35 level in overweight patients were significantly decreased than those in lean patients. Furthermore, Th1/Th2/Th17-related cytokines from overweight patients with RA showed the characteristic immunological features. Serum IL-6, IL-17 and TNF-α levels were positively correlated with BMI. However, serum IL-2, IL-4, IL-10 and IFN-γ concentrations were not correlated with BMI.

**Conclusions:**

Quantitative changes in serum IL-35 level were characteristic in overweight patients with RA. These findings indicate that IL-35 plays an important role in the development of RA and may prove to be a potential biomarker of active RA.

**Supplementary Information:**

The online version contains supplementary material available at 10.1186/s12865-021-00431-x.

## Background

Rheumatoid arthritis (RA) is characterized as a chronic inflammatory autoimmune disease which caused by multiple genetic and environmental factors. Some environmental factors including smoking and obesity has been demonstrated to significantly affect the prognosis of patients with RA. Obesity has also been confirmed as one of the most important environmental risk factors for RA. It is reported that obesity could prolong the disease course and give rise to poor responses to biological therapy [1, 2]. High disease activity is also observed in obese patients with early RA [3]. RA patients who have a high body mass index (BMI) were confirmed to have worse outcomes regarding to disease activity and comorbidities [4]. Previous studies have showed that obesity and inflammation are tightly linked, which contributes to the risk and severity of RA. The linkage between obesity and RA is being investigated. Multiple immune cells orchestrate and interplay in the development of RA. The underlying hypothesis is that white adipose tissue which secretes multiple pro-inflammatory and anti-inflammatory cytokines such as interleukin (IL)-6, tumor necrosis factor (TNF)-α could modulate immune response and inflammation, and store energy under conditions of caloric surplus [5–7].

IL-35, a novel cytokine, is a member of IL-12 family. It consists of the IL-12p35 subunit and IL-27subunit Epstein-Barr virus-induced 3 (EBI3, [8]). IL-35 is reported as an immunosuppressive cytokine and is mainly produced by regulatory T cells (Tregs), regulates immunological functions through inhibiting the inflammatory response [9]. IL-35 could induce the production of a subset of Tregs and Bregs, regulate their immuno-suppressive function and suppress the differentiation of Th17 cells. It could also upregulate the expression of osteoprotegerin and inhibit the expression of RANKL, thus inhibiting the progression of bone loss in collagen-induced arthritis (CIA) mice model. Furthermore, IL-35 inhibited proliferation and promoted apoptosis of FLS in CIA mice. Yokota et al. found that IL-35 significantly reduced the production of IL-4, IL-17 and TNF-α, and significantly increased the production of IL-2 and IL-10 [10]. IL-35 secretion is influenced by immune states. Studies demonstrated that injection of recombinant IL-35 can be used to protect against autoimmune diabetes [11]. Besides IL-35, various cytokines, both proinflammatory and anti-inflammatory, also have been analyzed in patients with RA, and the balance between the activities of these opposite cytokines lead to disease severity [12]. The imbalance between pro-inflammatory and anti-inflammatory cytokines responses favors the activation of autoimmunity and thereby joint destruction [13]. Considering the relationship between cytokines and RA symptoms of patients, multiple Th1/Th2/Th17-related cytokines were detected as the crucial biomarkers for patients with RA [14]. Modulation of the imbalance of pro-inflammatory and anti-inflammatory responses, particularly for Th1 and Th2 as well as their cytokine responses is a therapeutic strategy for retarding the pathogenic process of RA [15, 16].

In the previous study, the serum IL-35 level was compared between patients with RA and health controls, and some differences were identified, such as an increase of serum IL-35 level in patients with RA. In the current study, we analyzed this information in view of their relationship with BMI. Furthermore, Th1/Th2/Th17-related cytokines were also analyzed in order to explore the immunological features in overweight patients with RA.

## Results

### Characteristics of the study population

Patients were matched for age, gender, and BMI. Meanwhile, patients were categorized into three groups according to BMI (< 18.5, ≥18.5 to 25, > 25), as previously defined. A summary of RA patient profiles is listed in Table 1.

**Table 1:** Clinical parameters of RA and HCs

Characteristics	RA (***n*** = 81)	HCs (***n*** = 53)	***P*** value	RA patients(***n*** = 81)			***P*** value
				BMI < 18.5 (***n*** = 17)	18.5 ≤ BMI ≤ 25 (***n*** = 47)	BMI > 25 (***n*** = 17)	
***Demographics***							
Age, mean ± SE, years	50.0 ± 1.6	53.3 ± 2.8	0.67	41.6 ± 3.9	52.5 ± 2.1	52.1 ± 2.6	0.64
Sex (F/M)	72/9	50/3	0.88	18/1	41/5	13/3	0.54
***Lipid profiles***							
TC, mean ± SE, mmol/L	4.3 ± 0.1	4.2 ± 0.3	0.74	3.9 ± 0.2	4.4 ± 0.1	4.4 ± 0.3	0.13
TG, mean ± SE, mmol/L	1.1 ± 0.07	0.8 ± 0.1	0.55	0.8 ± 0.06	1.1 ± 0.1	1.3 ± 0.2	0.087
HDL-C, mean ± SE, mmol/L	1.2 ± 0.04	1.3 ± 0.02	0.34	1.2 ± 0.08	1.2 ± 0.07	1.0 ± 0.07	0.03
LDL-C, mean ± SE, mmol/L	2.6 ± 0.08	2.5 ± 0.1	0.48	2.3 ± 0.1	2.6 ± 0.1	2.9 ± 0.2	0.080
Apoplipoprotein A1, mean ± SE, g/L	1.3 ± 0.03	1.3 ± 0.05	0.93	1.3 ± 0.08	1.3 ± 0.05	1.2 ± 0.07	0.82
Apoplipoprotein B, mean ± SE, g/L	0.8 ± 0.02	0.9 ± 0.01	0.81	0.7 ± 0.04	0.8 ± 0.03	0.9 ± 0.07	0.015
***Disease characteristics***							
ESR, mean ± SE, mm/h	56.8 ± 4.5	–		56.7 ± 11.1	54.2 ± 6.1	63.1 ± 13.3	0.68
CRP, mean ± SE, mg/L	38.2 ± 5.0	–		40.4 ± 11.8	27.0 ± 4.3	55.8 ± 19.5	0.77
TJC, mean ± SE	8 ± 0.1	–		7 ± 0.1	9 ± 0.2	9 ± 0.1	0.81
SJC, mean ± SE	8 ± 0.2	–		7 ± 0.3	8 ± 0.2	9 ± 0.3	0.86
DAS28-ESR, mean ± SE	5.9 ± 0.2	–		5.5 ± 0.7	5.8 ± 0.3	6.0 ± 0.5	0.09
RF titers, mean ± SE, IU/mL	226.4 ± 41.5	–		331.1 ± 75.2	262.2 ± 63.2	113.6 ± 41.38	0.06
ACPA titers, mean ± SE, U	281.5 ± 14.9	–		279.1 ± 41.1	306.3 ± 20.4	256.3 ± 44.8	0.88

*Abbreviations: RA* rheumatoid arthritis, *HCs* healthy controls, *BMI* body mass index, *TC* total cholesterol, *TG* triglyceride, *HDL-C* high-density lipoprotein cholesterol, *LDL-C* low-density lipoprotein cholesterol, *ESR* erythrocyte sedimentation rate; CRP, C-reaction protein; TJC, tender joint count; SJC, swollen joint count; *VAS* visual analogue scale, *DAS28-ESR* disease activity score in 28 joints based on erythrocyte sedimentation rate, *RF* rheumatoid factor, *ACPA* anti-cyclic citrullinated peptide antibodies, *IQR* interquartile range, *SE* standard error

### Correlation between serum IL-35 levels and obesity

**Fig. 1:**
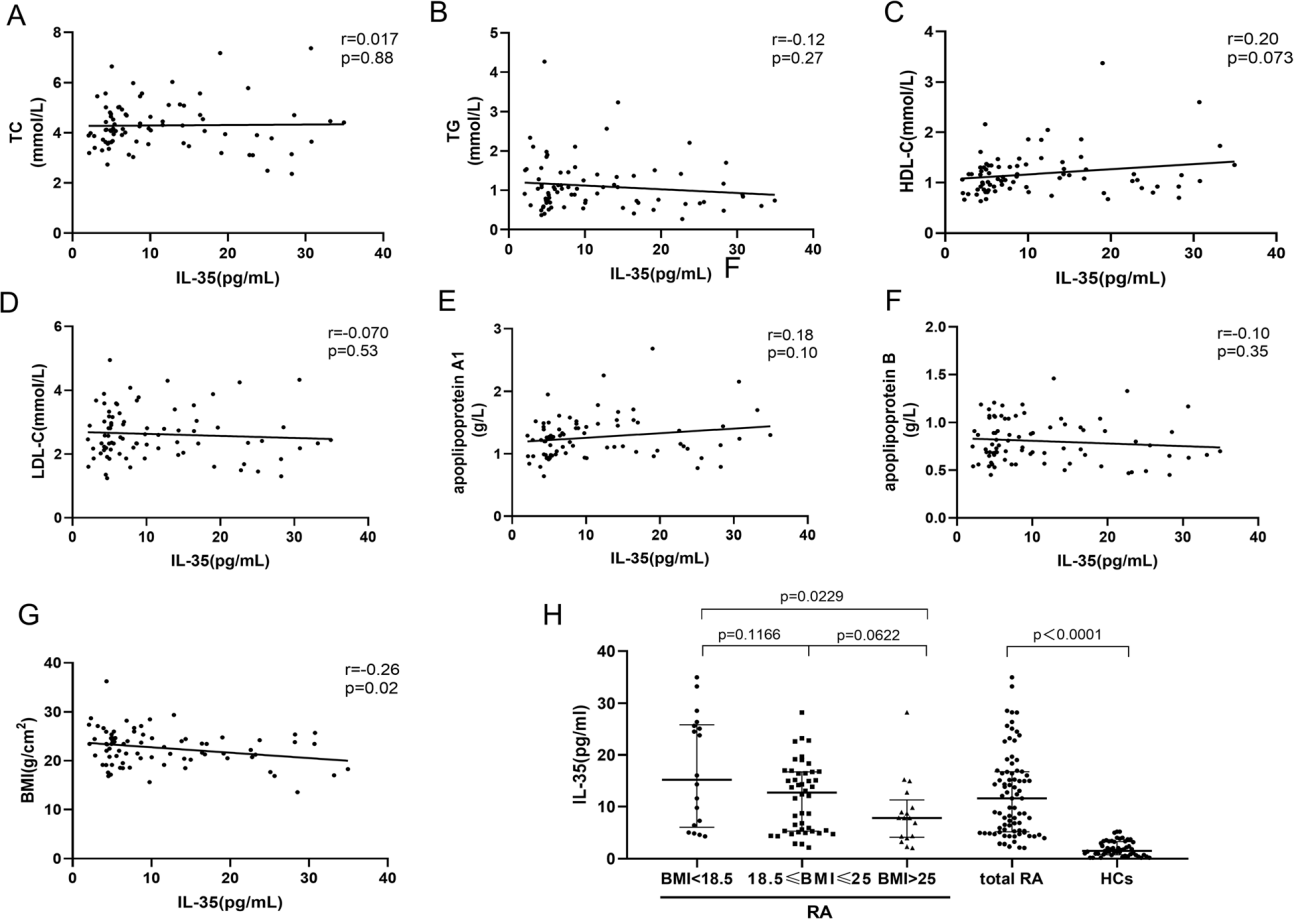
(**A**, **B**, **C**, **D**, **E**, **F**) Correlation between serum IL-35 levels and lipid profile including TC, TG, HDL-C, LDL-C, apoplipoprotein A1 and apoplipoprotein B;(**G**) Correlation between serum IL-35 levels and BMI; (**H**) Serum IL-35 levels in three groups categorized to BMI (< 18.5, ≥18.5 to 25, > 25). Abbreviation: TC, total cholesterol; TG, triglyceride; HDL-C, high-density lipoprotein cholesterol; LDL-C, low-density lipoprotein cholesterol; IL, Interleukin; BMI, body mass index

In current study, BMI and lipid profile including total cholesterol, triglycerides, LDL-C, HDL-C, apoplipoprotein A1 and apoplipoprotein B were used to explore the relationship between IL-35 and obesity in RA. The serum IL-35 level in total RA patients, three subgroups and healthy controls were presented in Table 2.We found that the serum IL-35 level in total patients with RA was significantly elevated compared to that in healthy controls (median (IQR), 11.6(5.1–16.8) pg/ml vs 1.5(0.8–3.2) pg/ml)(p<0.0001). Furthermore, serum IL-35 level was negatively correlated with BMI in patients with RA (*r* = − 0.26, *p* = 0.02). In addition, serum IL-35 level in overweight patients were significantly decreased than those in lean patients (*p* = 0.0229). However, IL-35 were not correlated with serum levels of lipids (*p*>0.05) (Fig. 1).

**Table 2:** Serum IL-35 level in RA patients and HCs

	RA (***n*** = 81)	HCs (***n*** = 53)	***P*** value	RA patients (***n*** = 81)		
				BMI < 18.5 (***n*** = 17)	18.5 ≤ BMI ≤ 25 (***n*** = 47)	BMI > 25 (***n*** = 17)
Serum IL-35 levels median (IQR), (pg/ml)	11.6(5.1–16.8)	1.5(0.8–3.2)	<0.0001	15.2(6.0–25.8)	12.7(5.3–16.7)	7.8(4.1–11.3)

*Abbreviation: RA* rheumatoid arthritis, *HCs* healthy controls, *BMI* body mass index, *IQR* interquartile range

### Correlations between Th1/Th2/Th17-related cytokines and BMI

Serum pro-inflammatory and anti-inflammatory cytokines were measured in patients with RA and were analyzed for correlation with BMI. Serum IL-6, IL-17A and TNF-α levels were positively correlated with BMI (*r* = 0.2292, *p* = 0.0067; *r* = 0.4761, *p* < 0.0001; *r* = 0.4500, *p* < 0.0001, respectively). However, serum IL-2, IL-4, IL-10 and IFN-γ concentrations were not correlated with BMI (*r* = − 0.1395, *p* = 0.0835; *r* = 0.1326, *p* = 0.2381; *r* = − 0.0571, *p* = 0.6126; *r* = 0.0699, *p* = 0.5353, respectively) (Table 3). The regression analysis to highlight correlations between BMI and DAS28, Th1/Th2/Th17 related cytokines was shown in the (Supplemental Fig. 1).

**Table 3:** Correlations between BMI and DAS28, Th1/Th2/Th17-related cytokines in patients with RA

	Coefficient	P
**DAS28**	0.2258	0.0427
**IL-2**	−0.1395	0.0835
**IL-4**	0.1326	0.2381
**IL-6**	0.2292	0.0067
**IL-10**	−0.0571	0.6126
**IL-17A**	0.4761	*p* < 0.0001
**IFN-γ**	0.0699	0.5353
**TNF-α**	0.4500	*p* < 0.0001

*Abbreviations: DAS28* disease activity score in 28 joints based on erythrocyte sedimentation rate; *IL* interleukin, *BMI* body mass index, *IFN* interferon, *TNF* tumor necrosis factor

### Cytokines expression in overweight patients with RA

**Fig. 2:**
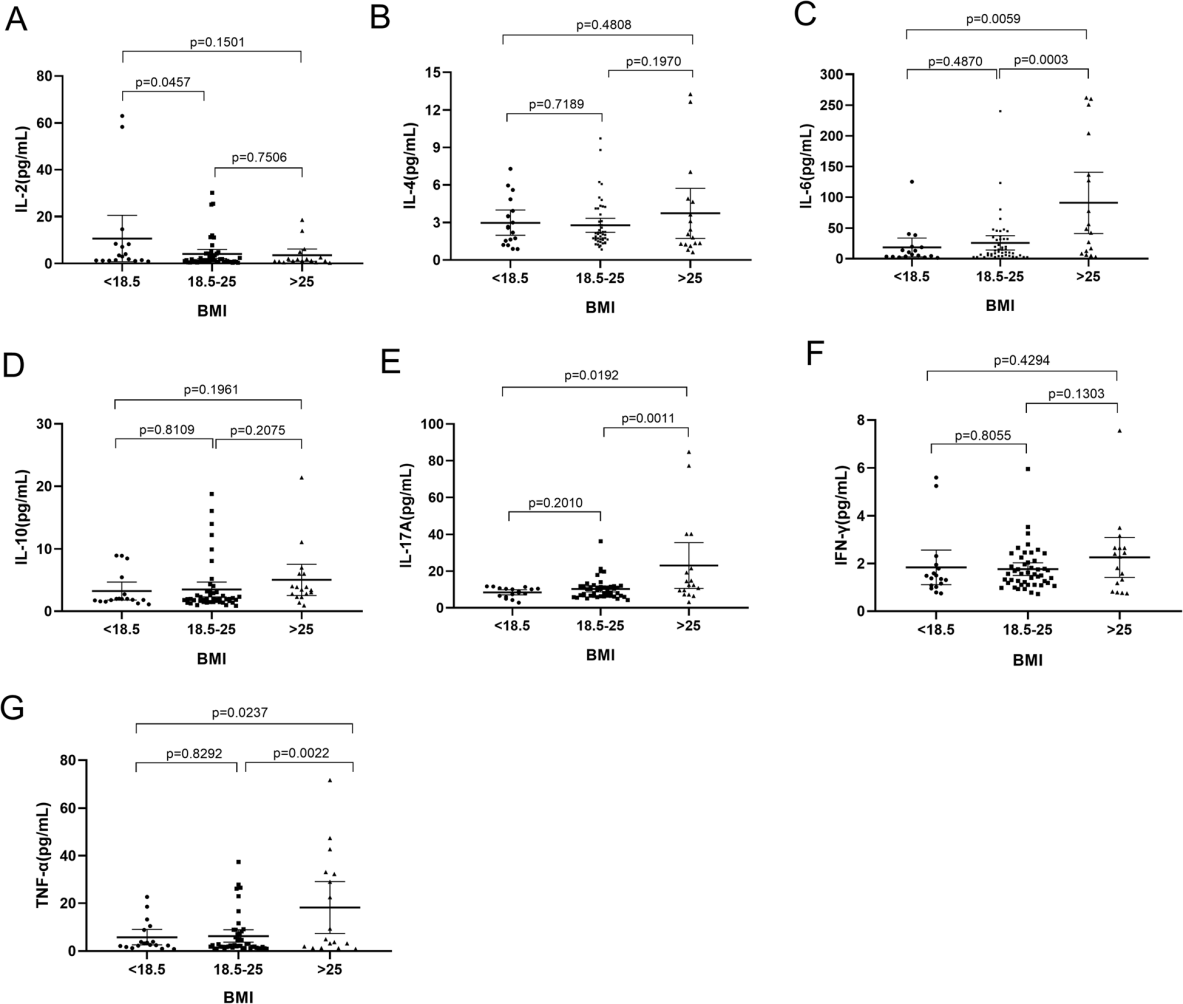
(**A**, **B**, **C**, **D**, **E**, **F**, **G**) Serum cytokines levels of IL-2, IL-4, IL-6, IL-10, IL-17A, IFN-γ and TNF-αin in three groups categorized to BMI (< 18.5, ≥18.5 to 25, > 25). Abbreviation: IL: interleukin; BMI: body mass index; IFN: interferon; TNF: tumor necrosis factor

The typical two-dimensional scatter diagrams of the frequency of Th1/Th2/Th17-Related Cytokines in patients with RA were shown in (Supplementary Fig. 2). In our study, serum levels of IL-6, IL-17 and TNF-α were found to be significantly higher (*p* = 0.0059, *p* = 0.00192, *p* = 0.0237, respectively) in the overweight group with RA (BMI>25) than in lean controls (BMI<18.5) (Fig. 2C, E, G). Moreover, no significant differences were observed in serum levels of IL-2, IL-4, IL-10 and IFN-γ between the 3 groups of patients with RA (Fig. 2A, B, D, F).

## Discussion

Obesity is a common condition in China and its global rise has contributed to subsequent morbidities. The levels of serum fibrinogen and other acute phase reactants, such as TNF-α, IL-6, and CRP were demonstrated to be increased in obesity [17]. This situation also leads to autoimmune diseases such as RA. Previous study confirmed that obesity involves in the progression and development of RA at different stages [18]. Cytokines are key regulators of inflammation which play an important role in inflammatory diseases via a complicated network of interactions. A better understanding of cytokine expression and related signaling pathways will help promote more effective treatment of chronic inflammatory diseases such as RA. In the current study we explored the correlation between cytokines and obesity by analyzing serum concentrations of multiple cytokines in lean, normal and overweight patients with RA.

The important finding from the current study is that BMI is associated with simultaneous production of serum IL-35 of overweight patients compared to lean patients. Our previous study showed that IL-35 was highly expressed in patients with RA and that serum IL-35 expression correlated negatively with the disease activity. This result was opposite to the findings of the Ning’s group [19]. This contradicting result could be explained by different sample size, disease duration and medical type in RA patients, which suggesting IL-35 might play different immunoregulatory role in different phases of RA. Previous studies have confirmed that IL-35 is preferentially produced by regular T cells (Tregs) during immunosuppression and can stimulate their activity. Tregs require IL-35 to maximize their regulatory activity and curb T cell proliferation, which efficaciously refrain the development of inflammation [20]. Furthermore, IL-35 was found as an anti-inflammatory cytokine which could inhibit the differentiation of Th17 cells in murine models of RA [21]. We previously found that serum levels of IL-35 negatively correlated with disease activity in patients with RA [22]. Meanwhile, serum IL-35 levels were significantly increased in response to tofacitinib in patients with RA, which suggesting IL-35 play an important role in the immunomodulation of RA [23]. In the state of obesity, the imbalance of Th1, Th2, Th17 and Tregs are observed. The results showed that serum level of IL-17 was found to be significantly higher in the overweight group with RA than in lean controls which coincided exactly with experimental results of Hirofumi Shoda, et al, which demonstrating quantitative and qualitative changes in Th17 cells were characteristic in overweight patients with RA [24]. These alterations suggest a loss of T cell homeostasis, which may lead to local tissue and systemic inflammation and immunity in RA. Recent studies have confirmed that T cells are mediated in adipose tissues and may lead to obesity-induced inflammation [25]. Due to the anti-inflammatory role of IL-35 and its selective activities to proliferate regulatory T cells and suppress Th17 cells, it could postulate that the negative correlation between IL-35 and BMI due to the negative feedback immune response activated by pro-inflammatory molecules, which could inhibit excessive autoimmune response.

In order to analyze the potential regulators involved with the altered lymphocyte activation in overweight patients with RA, serum concentrations of Th1/Th2/Th17-realted cytokines were measured. Obesity is characterized by a state of chronic low-grade inflammation called meta-inflammation in which the immune cells, especially monocytes, are induced, infiltrate the adipose tissue and become differentiated as resident adipose tissue macrophages (ATMs). ATMs are classified by the expression of M1 or proinflammatory markers including IL-6 and TNF-α [26]. Therefore, it could be explicable that serum levels of IL-6 and TNF-α are higher in obese patients with RA than those of lean patients with RA. Furthermore, changes in the IL-35 with changes in the IL-17 is rational which may be due to the suppressive role of IL-35 which affect the proliferation and differentiation of Th17 as the producers of IL-17. Recent studies have demonstrated that obesity selectively stimulate the secretion of IL-17 producing CD4 + T (Th17) cells in adipose tissues, aggravating autoimmune response in mice model [27]. In obese collagen-induced arthritis mice model, which is a new animal model of RA, indicates the amplified inflammatory state, especially through Th17 deviation. The number of Th17 cells and IL-17 mRNA expression of the splenocytes were elevated in obese CIA mice compared with those of lean counterpart [28]. However, other non-significant relationships between BMI and serum concentrations of IL-2, IL-4, IL-10 and IFN-γ could not be explained that obesity induce no changes in the expression of above cytokines, which might be related to the determination of above cytokines in the local joint tissue-not in the serum. For more confirmation, evaluation the serum level of IL-2, IL-4, IL-10 and IFN-γ in synovial fluid and fibroblast-like synoviocytes in patients with RA and in healthy controls.

## Conclusions

BMI was closely linked to chronic inflammation in patients with RA, and quantitative change of IL-35 were observed in overweight patients with RA. We speculated that the overweight patients with RA were a subgroup of patients with RA who had the unique immunological features. The role of IL-35 should be further investigated in RA, especially overweight patients in RA. A better understanding of the arthritis-obesity interaction will help us to improve a personalized management directed against both the disease and its associated comorbidities.

## Methods

### Patients

A total of 81 patients with RA and 53 healthy controls (HCs) at the First Affiliated Hospital of China Medical University and the Shengjing hospital of China Medical University were recruited for this study. The recruited patients all met the American Colleague of Rheumatology criteria for RA [29]. All patients did not receive any therapy with glucocorticoids and/or immunosuppressants. Body mass index (< 18.5, ≥18.5 to 25, > 25) was classified as previously defined. Blood tests for erythrocyte sedimentation rate (ESR) and C-reactive protein (CRP) were measured by Westergren method, immune transmission turbidity method, respectively. Rheumatoid factor (RF) (cut-off value of 19 IU/ml) and anti-cyclic citrullinated peptide antibodies (ACPA) (cut-off value of 20 U) were assessed by immunoturbidimetric assays and chemiluminescence analysis, respectively. Each patient’s swollen joint count (SJC) and tender joint count (TJC) were assessed. Written informed consent was provided by all subjects, and the study was approved by the ethics committee of the First Affiliated Hospital of China Medical University and Shengjing Hospital of China Medical University and was conducted according to the Declaration of Helsinki.

### Detection of serum IL-35

Blood samples were acquired from the recent-onset RA patients. Serum IL-35 levels were measured using Enzyme Linked Immunosorbent Assay (ELISA) kits (eBioscience, San Diego, CA, USA) according to the manufacturer’s protocol. Briefly, Add 100 μL of sample or standards in reagent diluent (1:1) per well. Cover with an adhesive strip and incubate 2 h at room temperature. Repeat the aspiration/wash as previous plate preparation. Add 100 μL of the Detection Antibody, diluted in reagent diluent, to each well. Cover with a new adhesive strip and incubate 2 h at room temperature. Repeat the aspiration/wash as previous plate preparation. Add 100 μL of the working dilution of Streptavidin-HRP to each well. Cover the plate and incubate for 20 min at room temperature. Avoid placing the plate in direct light. Repeat the aspiration/wash as previous plate preparation. Add 100 μL of substrate solution to each well. Incubate for 20 min at room temperature. Avoid placing the plate in direct light. Add 50 μL of Stop Solution to each well. Gently tap the plate to ensure thorough mixing. The optical density was measured at 450 nm using an automatic ELISA reader.

### Determination of Th1/Th2/Th17-related cytokine levels

Serum cytokines, which were representative of Th1/Th2/Th17-related cytokines including IL-2, IL-4, IL-6, IL-10, IL-17A, INF-γ, TNF-α levels, were measured using Flowcytometry assay (BD™ Cytometric Bead Array Human Th1/Th2/Th17 Cytokine Kit) according to the manufacturer’s protocol. BD FACSVerse system were used for the detection and analysis of the serum levels of the cytokines IL-2, IL-4, IL-6, IL-10, IL-17A, IFN-γ, and TNF-α.

### BMI and lipid profile assessment

BMI was assessed as the weight in kilograms divided by height in meters squared. It was categorized into three groups according to the definition of the World Health Organization (WHO): underweight (< 18.5 kg/m^2^), normal weight (18.5-<25 kg/m^2^), overweight and obese (≥25 kg/m^2^).

Lipid profile included total cholesterol (TC), triglyceride (TG), low-density lipoprotein cholesterol (LDL-C), high-density lipoprotein cholesterol (HDL-C), apoplipoprotein A1, and apoplipoprotein B. All lipids were measured using the automatic biochemical analyzer (Abbott ARCHITECT ci16200, American) at our hospital’s laboratory. The level of serum TC was measured using cholesterol oxidase method (Hitachi Chemical Diagnostics Systems, No 061039). The level of serum TG was measured using GB&Enzyme method (Hitachi Chemical Diagnostics Systems, No 0610926). The level of serum LDL-C was measured using selective melt method (Hitachi Chemical Diagnostics Systems, No 061036). The level of serum HDL-C was measured using chemical modification enzyme method (Hitachi Chemical Diagnostics Systems, No 061077). And the levels of serum apoplipoprotein A1 and apoplipoprotein B were measured using turbidimetric inhibition immuno assay (Dia System, No, 50,244,100/26467, 50,244,140/26807).

### Statistical analysis

All data are presented as mean ± standard error (SE) or median (interquartile range (IQR)) if continuous and as counts and percent if categorical. Differences between groups were tested with one-way analysis of variance (ANOVA), followed by a Turkey’s multiple comparisons post-test. χ^2^, *t-*tests and logistic regression models were used to compare the study groups and to explore the association between obesity and serum cytokines in RA. All analyses were performed using SPSS 17.0 (SPSS Inc., Chicago, IL), and GraphPad prism 7 software. Differences of *p* < 0.05 were considered significant.

## Supplementary Information

**Additional file 1: Supplementary Figure 1.** (A-H) Correlation between BMI and DAS28, IL-2, IL-4, IL-6, Iil-17A, IFN-γ and TNF-α. Abbreviations: DAS28: disease activity score in 28 joints based on erythrocyte sedimentation rate; IL: interleukin; BMI: body mass index; IFN: interferon; TNF: tumor necrosis factor.

**Additional file 2: Supplementary Figure 2.** (A-D) The typical two-dimensional scatter diagrams of the frequency of Th1/Th2/Th17-Related Cytokines in patients with RA.

## Data Availability

The data used to support the findings of this study are available from the corresponding author upon request.

## Abbreviations

RA: rheumatoid arthritis
BMI: body mass index
IL: interleukin
TNF: tumor necrosis factor
IFN: interferon
EBI3: Epstein-Barr virus-induced 3
Tregs: regulatory T cells
CIA: collagen-induced arthritis
HCs: healthy controls
CRP: C-reactive protein
ESR: erythrocyte sedimentation rate
RF: rheumatoid factor
ACPA: anti-cyclic citrullinated peptide antibodies
SJC: swollen joint count
TJC: tender joint count
VAS: visual analogue scale
DAS28-ESR: disease activity score in 28 joints based on erythrocyte sedimentation rate
IQR: interquartile range
SE: standard error
ELISA: Enzyme Linked Immunosorbent Assay
TC: total cholesterol
TG: triglyceride
LDL-C: low-density lipoprotein cholesterol
HDL-C: high-density lipoprotein cholesterol
