# Supplementary figures and images for: Serum IL-35 is decreased in overweight patients with rheumatoid arthritis: its correlation with Th1/Th2/Th17-related cytokines
Source: BMC Immunol. 2021 Jun 27;22:42. doi: 10.1186/s12865-021-00431-x (PMC8237483; doi:10.1186/s12865-021-00431-x)

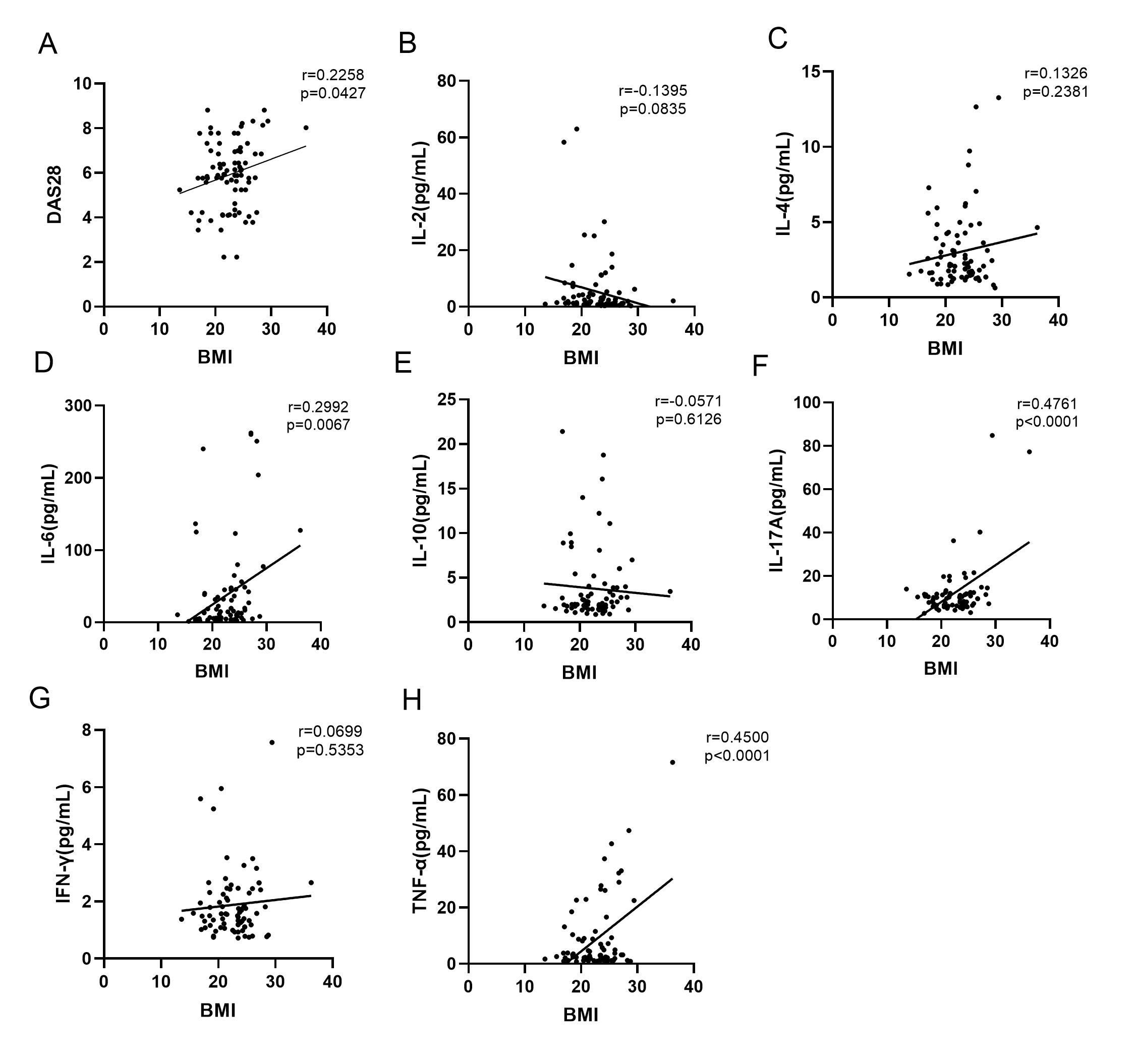

Supplement: Supplementary file 1 — Additional file 1: Supplementary Figure 1. (A-H) Correlation between BMI and DAS28, IL-2, IL-4, IL-6, Iil-17A, IFN-γ and TNF-α. Abbreviations: DAS28: disease activity score in 28 joints based on erythrocyte sedimentation rate; IL: interleukin; BMI: body mass index; IFN: interferon; TNF: tumor necrosis factor. [file 12865_2021_431_MOESM1_ESM.jpg]

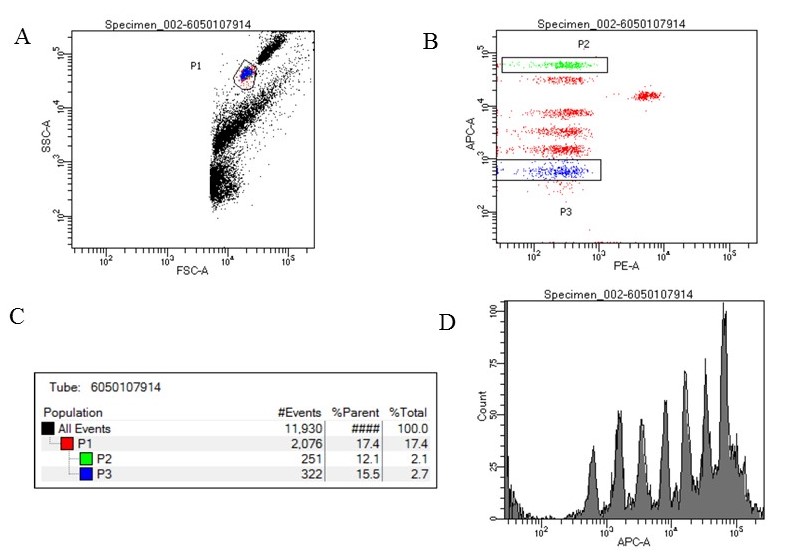

Supplement: Supplementary file 2 — Additional file 2: Supplementary Figure 2. (A-D) The typical two-dimensional scatter diagrams of the frequency of Th1/Th2/Th17-Related Cytokines in patients with RA. [file 12865_2021_431_MOESM2_ESM.jpg]
